# Treatment of Relapsed or Refractory Diffuse Large B-Cell Lymphoma: New Approved Options

**DOI:** 10.3390/jcm13010070

**Published:** 2023-12-22

**Authors:** Alejandro Martín García-Sancho, Almudena Cabero, Norma C. Gutiérrez

**Affiliations:** Hematology Department, University Hospital of Salamanca, IBSAL (Instituto de Investigación Biomédica de Salamanca), CIBERONC (Centro de Investigación Biomédica en Red en Cáncer ), University of Salamanca, 37007 Salamanca, Spain; acaberoma@saludcastillayleon.es (A.C.); normagu@usal.es (N.C.G.)

**Keywords:** diffuse large-B cell lymphoma, relapsed or refractory DLBCL, CAR-T cell therapy, polatuzumab vedotin, tafasitamab, autologous stem cell transplantation

## Abstract

Overall, around 40% of patients with diffuse large B-cell lymphoma (DLBCL) have refractory disease or relapse after the first line of treatment. Until relatively recently, the prognosis of patients with relapsed or refractory DLBCL was very poor and treatment options were very limited. In recent years, several novel therapies have been approved that provide more effective options than conventional chemotherapy and that have manageable toxicity profiles. CAR-T cell therapy has become the new standard treatment for patients with refractory or early relapsed DLBCL, based on the positive results of the phase 3 ZUMA-7 and TRANSFORM clinical trials. This review addresses the role of CAR-T therapy and autologous stem cell transplantation in the treatment of these patients and other approved options for patients who are not candidates for transplant, such as the combinations of polatuzumab vedotin with bendamustine and rituximab, and tafasitamab with lenalidomide.

## 1. Introduction

Overall, around 40% of patients with diffuse large B-cell lymphoma (DLBCL) have refractory disease (15–20%) or relapse (20–30%) after the first line of treatment [1]. Before starting the second line, it is recommended to repeat the tumor biopsy, since positron-emission tomography (PET) may give false-positive results in patients who do not achieve metabolic complete remission (CR) after the first line (in this case, also repeat PET at 6–12 weeks if it is not feasible to perform a biopsy), or to exclude other diseases (such as tuberculosis, sarcoidosis, fungal infection, carcinoma, etc.) or histological changes in the case of patients in relapse [2].

Until relatively recently, the prognosis of patients with relapsed or refractory (R/R) DLBCL was very poor and treatment options were very limited. In recent years, several novel therapies have been approved that provide more effective options than conventional chemotherapy and that have manageable toxicity profiles. In the present review, we address the role of autologous stem cell transplantation (ASCT) and newly approved and established options for the treatment of patients with refractory or relapsed (R/R) DLBCL, such as CD19-targeted chimeric antigen receptor (CAR) T-cells, polatuzumab vedotin combined with rituximab and bendamustine (pola-RB), and tafasitamab combined with lenalidomide. Other more recently approved options, such as bispecific antibodies and loncastuximab tesirine, are reviewed in another article in this special issue.

## 2. Autologous Stem Cell Transplantation

Until recently, the established treatment of choice for patients with R/R DLBCL has been the administration of a platinum-based salvage regimen followed by ASCT. The superiority of ASCT over consolidation chemotherapy in patients with chemosensitive relapsed aggressive lymphoma was established by the randomized PARMA study in the 1990s [3] (Table 1). Although there are no randomized studies in the rituximab era, ASCT continues to be considered one of the standard options for these patients due to its curative potential [4]. The usual approach is to administer 2–3 cycles of the salvage regimen and, if at least a partial response (PR) is achieved, proceed with ASCT. Under these circumstances, the main prognostic factor is the lymphoma status at the time of transplant, since patients in metabolic CR have significantly better results than patients in PR, with progression-free survival (PFS) of 72–87% vs. 18–49% in non-randomized studies [5,6,7], while ASCT is not indicated for patients with chemorefractory disease (stable disease or progression after the salvage regimen) [4].

### 2.1. Salvage Regimens Prior to ASCT

Nowadays, with the use of highly effective first-line regimens that combine rituximab and chemotherapy, patients who are refractory or who relapse are more difficult to rescue with conventional immunochemotherapy. This fact was documented in a multicenter retrospective study by the GELTAMO group, which analyzed 163 patients treated with R-ESHAP (rituximab, etoposide, cytarabine, cisplatin, and methylprednisolone); patients pre-treated with rituximab had significantly worse PFS (17% vs. 57% at 3 years) and overall survival (OS) (38% vs. 67% at 3 years) [16].

To determine which is the best salvage regimen for patients who are candidates for ASCT, three phase 3 comparative studies have been carried out in the rituximab era (Table 1):

- In the CORAL study, 396 patients with R/R DLBCL were randomized to receive R-DHAP (rituximab, dexamethasone, ara-C, cisplatin) or R-ICE (rituximab, ifosfamide, carboplatin, etoposide), followed by ASCT in the chemosensitive patients. The efficacy of the two regimens was similar (Table 1), but R-DHAP had greater incidences than R-ICE of thrombocytopenia (platelet transfusions in 57% vs. 34% of patients) and grade 3–4 renal toxicity (6% vs. 1%) [8].
- In the Canadian LY.12 trial, 619 patients with R/R aggressive lymphoma (71% DLBCL) were randomized to receive (R)-GDP (rituximab, gemcitabine, dexamethasone, and cisplatin) or (R)-DHAP followed by ASCT. Again, the efficacy was similar (Table 1), but R-DHAP was associated with higher rates of hospitalization (99% vs. 47%), grade 3–4 toxicity (61% vs. 47%), febrile neutropenia (23% vs. 9%), platelet transfusions (47% vs. 31%) and treatment-related deaths (6 vs. 2 patients) [9].
- In the ORCHARRD trial, replacing rituximab with ofatumumab was not associated with any significant benefits (Table 1 and Table 2) [10].

In summary, there is no salvage regimen that has demonstrated superiority over the others in terms of efficacy, and all of them have shown poor results in patients pre-treated with rituximab (Table 2), with response rates between 40 and 50%, transplant rates less than 50% and long-term PFS around 20–25%. The choice of salvage regimen will depend on the clinical experience and preferences of the center and the toxicity profile of each regimen.

### 2.2. Prognostic Factors in the Context of R/R DLBCL

The status of lymphoma at the time of receiving the salvage regimen is a key prognostic factor. Thus, patients with primary refractory disease (progression or stable disease with first-line treatment) rarely respond to second line regimens (17% PR, 3% CR, according to the SCHOLAR-1 meta-analysis) [19], and patients with early relapse have worse outcomes than patients with late relapse (>1 year from diagnosis or from the end of the first line), with PFS at 2 years of 10–20% vs. 40–50% [8,10]. In this sense, the retrospective REFINE study analyzed 331 patients with either primary refractory DLBCL or with early progression (in the first 6 months after completing first-line treatment). This study described prognostic factors of “ultra-high risk” as the presence of progression during first-line treatment, MYC translocation, and National Comprehensive Cancer Network International Prognostic Index (NCCN-IPI) of intermediate-high or high risk. The presence of any of these three factors was associated with very low chances of survival (2-year OS of 13.6%). In a separate analysis of the cohort of patients who received ASCT (*n* = 132), the presence of two of these three ultra-high-risk factors was associated with very high rates of ASCT failure (2-year OS of 10.7%) [20].

Other studies have also shown the prognostic value of the International Prognostic Index (IPI) in this context [8,10,16]. In the CORAL study, 3-year event-free survival (EFS) was 18% vs. 40% in patients with an age-adjusted IPI of 0–1 vs. 2–3 [8]. Regarding biological prognostic factors, a subanalysis of the CORAL study revealed that patients with MYC rearrangement had worse outcomes with any of the salvage regimens (OS of 29% vs. 62% at 4 years) [21]. Another retrospective study (*n* = 117 patients) evaluated the prognostic impact of the presence of a double hit (defined as rearrangements of MYC and BCL2 and/or BCL6) or double expression of MYC and BCL2 on the outcomes of ASCT in patients with R/R DLBCL. The presence of a double hit was associated with worse PFS outcomes (28% vs. 57% at 4 years, *p* = 0.013) and OS (25% vs. 61% at 4 years, *p* = 0.002), and remained as an independent prognostic factor in the multivariate analysis. Notably, the estimated 4-year PFS was 0% in patients who simultaneously had a double hit and double expression [22].

Therefore, the strategy of platinum-based salvage treatment followed by ASCT may be curative for some patients with R/R DLBCL. However, high-risk groups, such as primary-refractory or early-relapsed patients, adverse IPI or double-hit cases, have very poor outcomes and require other treatment modalities.

## 3. CAR-T Cell Therapy

Immunotherapy with anti-CD19 CAR-T cells represents the new standard of treatment for patients with refractory DLBCL. Three CAR-T cell products, axicabtagene ciloleucel (axi-cel), tisagenlecleucel (tisa-cel), and lisocabtagene maraleucel (liso-cel), have been approved by the European Medicines Agency (EMA) and the Food and Drug Administration (FDA) for the treatment of patients with R/R large B-cell lymphomas (LCBGs) pre-treated with two lines. Axi-cel and liso-cel also have been approved as a second line for primary refractory patients or those who relapse early, based on the clinical trials that we will discuss below.

### 3.1. CAR-T Therapy as Third Line or Subsequent Treatment

Axi-cel, tisa-cel and liso-cel have been associated with very high response rates (52–83%, CR 40–58%) in phase 2 clinical trials [23,24,25] of patients with DLBCL who had been pre-treated with at least two lines, the vast majority of whom had chemorefractory disease (Table 3). Most importantly, prolonged remissions occurred in approximately 30–40% of patients, with a plateau in survival curves after long-term follow-up (5 years for axi-cel [26], 3 years for tisa-cel [27] and 2 years for liso-cel [28]), indicating the curative potential of this modality of treatment. Treatment-related toxicity is very high in the early phase after administration, mainly arising from cytokine release syndrome (CRS) (42–93% of patients, grades 3–4 in 2–22%) and immune-effector cell-associated neurotoxicity syndrome (ICANS) (21–64% of patients, grades 3–4 in 10–28%) [16,17,18]. It is reversible in the vast majority of cases if the patient receiving CAR-T therapy is in a good general condition and has adequate organ function. It is also worth highlighting the frequent appearance of late cytopenias, beyond day 30, which can be severe in 30–40% of patients, as well as the aplasia of B-cells and hypogammaglobulinemia, which lead to severe immunosuppression and an elevated risk of serious or even fatal infections (grades 3–5 in 12–28% of patients treated in clinical trials) [23,24,25].

Despite its complexity, CAR-T cell therapy has become the standard treatment for these patients and many real-world studies have already been published that, in general, reproduce the efficacy results of clinical trials [29,30,31,32,33,34,35,36,37,38], even though the majority of patients had not met the eligibility criteria for the trials, mainly due to their comorbidities [31,38]. Non-relapse mortality (NRM) is around 1% and 5% during the first month and the first year, respectively, with infections being the main cause of death [39]. The incidence of severe CRS and ICANS in real-life studies is slightly lower than that described in clinical trials, possibly due to the earlier use of tocilizumab and steroids. The incidence is also lower with constructs containing 41BB as a co-stimulatory domain (tisa-cel and liso-cel) compared with CD28 (axi-cel), especially the incidence of severe ICANS (around 5–8% vs. 15–30%) [29,32,33,37,38]. Tisa-cel and liso-cel could therefore be administered on an outpatient basis. On the other hand, the time described in the studies between apheresis and infusion is longer for tisa-cel than for axi-cel [29,36,37] and several indirect comparisons suggest that axi-cel is more effective than tisa-cel [36,40]. Therefore, when choosing between the three products, the risk/benefit balance and the characteristics of the disease must be assessed individually for each patient [41].

Significant challenges remain in the use of CAR-T therapy in heavily pre-treated patients. First, given the need for rapid treatment in patients with aggressive chemorefractory disease, delays in product manufacturing may affect patient eligibility, so “bridging” therapy with steroids, radiotherapy or immunochemotherapy is needed in most cases to control the disease until the product can be infused [29,32,34]. In this sense, patients whose general condition has deteriorated, or who have a large tumor volume or high LDH levels have worse results with CAR-T therapy [29,30,32,42,43]. On the other hand, another important barrier to improving results is the possible intrinsic dysfunction of T cells in the infused product. The presence of “exhausted” T cell phenotypes limits product expansion and persistence, and regulatory CAR-T cells have been associated with treatment failure [44,45]. New designs and constructs are being investigated, such as those directed against several targets, those that use new platforms for rapid manufacturing, CAR-NK cells or allogeneic CARs, which will possibly expand the possibilities of this treatment modality in the future [41].

**Table 3:** Phase 1–2 clinical trials based on CAR-T cell therapy, polatuzumab vedotin or tafasitamab in patients with relapsed or refractory LBCL.

Patient Characteristics	Design	Efficacy	AESIs (Grade ≥3)
**CAR-T Cell Therapy in Patients Pre-Treated with ≥2 Lines**			
ZUMA-1 [23,26,46] DLBCL, 76%; PMBCL, 8%; tFL, 16% Median age 58 (23–72) y 100% refractory ^1^ 70% ≥ 3 prior therapies	Bridging chemo not allowed (only steroids) Cy-Flu conditioning Axi-cel 2 × 10^6^/kg Turnaround ^2^ NR Phase 2 (PE: ORR)	*n* = 101 out of 111 treated ORR, 82%; CR, 54% EFS (2 y), 38%; median, 5.7 mo OS (5 y), 43%; median 25.8 mo	CRS ^3^, 11% Neurological, 33% Infections, 28% Prolonged cytopenias ^5^, 38%
JULIET [24,27] DLBCL, 80%; HGBCL, 15%, tFL, 18% Median age 56 (22–76) y 55% refractory Median 3 (IQR 2–3) prior lines	Bridging chemo allowed (90%) Cy-Flu conditioning Tisa-cel 5 × 10^8^/kg Turnaround ^5^ 54 days Phase 2 (PE: ORR)	*n* = 115 out of 167 treated ORR, 53%; CR, 39% PFS (3 y), 31%; median, 2.9 mo OS (3 y), 36%; median 11.1 mo	CRS ^4^, 23% Neurological, 11% Infections, 19% Prolonged cytopenias ^5^, 41%
TRANSCEND [25,28] DLBCL, 51%; PMBCL, 6%; HGBCL, 13%, tFL, 29%; FLG3B, 1% Median age 63 (54–70) y 67% refractory ^1^ Median 3 (IQR 2–4) prior lines	Bridging chemo allowed (59%) Cy-Flu conditioning Liso-cel 100 × 10^6^/kg Turnaround ^2^ NR Phase 1/2 (PE: ORR)	*n* = 270 out of 344 treated ORR, 73%; CR, 53% PFS (2 y), 41%; median, 6.8 mo OS (2 y), 50%; median 27.3 mo	CRS ^3^, 2% Neurological, 10% Infections, 12% Prolonged cytopenias ^5^, 30%
**Clinical trials in patients non-candidates to ASCT**			
GO29365 [47,48] Median age 69 (24–94) y DLBCL, 95%; HGBCL, 3% Median 2 (1–7) prior lines 64% primary refractory 76% refractory to last line	Pola-RB × 6 cycles Phase 2 (PE, CR rate) Randomized (*n* = 40) and extension cohorts (*n* = 106)	*n* = 106 (extension cohort) ORR, 41%, CR, 39% PFS, median, 6.6 mo OS (1 y), 50%; median 12.5 mo	Pooled (*n* = 151) Neutropenia, 32% Thrombocytopenia, 20% Infections, 22% PN, 2%
L-MIND [49,50,51] Median age 72 (41–86) y DLBCL, 100%; HGBCL excluded Median 2 (1–4) prior lines Primary refractory excluded 44% refractory to last line	Induction: Tafasitamab + lenalidomide × 12 cycles Maintenance: Tafasitamab until PD Phase 2 (PE, ORR)	*n* = 80 ORR, 60%, CR, 43% PFS (1.5 y), 46% median, 11.6 mo OS (1.5 y), 64%; median 33.5 mo	Neutropenia, 48% Thrombocytopenia, 17% Febrile neutropenia, 12% Infections, 26% Rash, 9%
PILOT [52] DLBCL, 54%; HGBCL, 30%, tFL, 15%; FLG3B, 2% Median age 74 (53–84) y 54% refractory, 21% ER Only 1 prior line	Bridging therapy (84%) Cy-Flu conditioning Liso-cel 100 × 10^6^/kg Turnaround ^5^ 53 days Phase 2 (PE: ORR)	*n* = 61 out of 74 treated ORR, 80%; CR, 54% PFS (1 y), 46%; median, 9.0 mo OS (1 y), 70%; median NE	CRS ^3^, 2% Neurological, 5% Infections, 7% Prolonged cytopenias ^5^, 30%
ALYCANTE [53] DLBCL, 84%; HGBCL, 10%, tFL, 2%; FLG3B, 2% Median age 70 (49–81) y 55% refractory, 45% ER Only 1 prior line	Bridging therapy (52%) Cy-Flu conditioning Axi-cel 2 × 10^6^/kg Turnaround ^5^ 41.5 days Phase 2 (PE: CR)	*n* = 62 out of 69 treated ORR, 76%; CR, 71% PFS (1 y), 49%; median, 11.8 mo OS (1 y), 78%; median NE	CRS ^3^, 8% Neurological, 14% Infections, 39% Prolonged cytopenias ^5^, 37%

^1^ SCHOLAR-1 criteria [12]; ^2^ Median time from inclusion to CAR T infusion; ^3^ Lee criteria [54]; ^4^ University of Pennsylvania grading scale [55]; ^5^ Beyond day 30. Abbreviations: AESIs, adverse events of special interest; axi-cel, axicabtagene ciloleucel; CAR-T, chimeric antigen receptor T-cells; CR, complete response; CRS, cytokine release syndrome; CT, chemotherapy; Cy-Flu, cyclophosphamide, fludarabine; DLBCL, diffuse large B-cell lymphoma; EFS, event-free survival; ER, early relapse (≤12 months); FLG3b, follicular lymphoma grade 3B; HGBCL, high-grade B-cell lymphoma; LCBG, large B-cell lymphoma; liso-cel, lisocabtagene maraleucel; m, months; overall survival; NE, not estimable (not reached); NR, not reported; ORR, overall response rate; PD, progressive disease; PE, primary endpoint; PMBCL, primary mediastinal large B-cell lymphoma; PN, peripheral neuropathy; Pola-BR, polatuzumab vedotin, bendamustine, rituximab; PR, partial response; R, rituximab; R/R, refractory or relapsed; tFL, transformed follicular lymphoma; tisa-cel, tisagenlecleucel; y, years.

### 3.2. CAR-T Cell Therapy as Second-Line Treatment

As shown in Table 1, the randomized phase 3 clinical trials ZUMA-7, BELINDA and TRANSFORM separately compared CAR-T therapy (axi-cel, tisa-cel and liso-cel, respectively), with standard second-line treatment (2–3 cycles of salvage immunochemotherapy chosen by the investigator from among several regimens defined in the protocol, followed by ASCT if a PR or CR is achieved), in patients eligible for ASCT with refractory DLBCL or in early relapse (during the first year following completion of first-line treatment):

- In the ZUMA-7 trial [11,12], axi-cel gave superior results to those of the standard treatment when the primary endpoint was analyzed, EFS, which was evaluated by an independent committee, and was defined as death, progression, initiation of a new line of treatment or stable disease as the best response on day +150. After a median follow-up of 24.9 months, the estimated EFS at 2 years was 41% vs. 16%, respectively (hazard ratio [HR], 0.40; 95% confidence interval [CI], 0.31–0.51, *p* < 0.001). Furthermore, in a recent follow-up update (median 47.2 months), better OS was also observed in the axi-cel arm, with 4-year estimates of 55% vs. 46%, respectively (HR, 0.73; 95% CI, 0.54–0.98; *p* = 0.03). The vast majority of patients had grade 3 or higher adverse events (91% and 83% in the axi-cel and standard treatment arms, respectively). In patients receiving axi-cel, 6% and 21%, respectively, had CRS and ICANS grade ≥3, although there were no deaths related to these events.
- The BELINDA trial [13] found no significant differences between tisa-cel and standard treatment in the analysis of the primary objective, EFS, as evaluated by an independent committee and defined as progression or stable disease from week 12, or death at any time. After a median follow-up of 10 months, the median EFS was 3 months in both arms (HR, 1.07; 95% CI, 0.82–1.40, *p* = 0.61). There were also no differences in the ORR (46.3% vs. 42.5% in the experimental and standard arms, respectively).
- Finally, in the TRANSFORM trial [14,15], liso-cel was superior to standard treatment with respect to the primary objective, EFS, which was evaluated by an independent committee and defined as death, progression, initiation of a new line of treatment or not achieving response at 9 weeks after randomization. After a median follow-up of 17.5 months, the median EFS had not been reached in the liso-cel arm and was 2.4 months in the standard treatment arm (HR, 0.36; 95% CI, 0.2–0.52, *p* < 0.001). A trend towards better OS was also observed in the liso-cel arm (73% vs. 61% at 18 months), although the difference was not statistically significant (HR, 0.72; 95% CI, 0.44–1.18; *p* = 0.0987). In patients receiving liso-cel, only 1% and 4%, respectively, had grade 3 CRS and ICANS, with no grade 4 or 5 events observed.

Although there may be intrinsic differences in the efficacy of the three products, it must also be borne in mind that the designs of the three clinical trials differ substantively, which could largely explain the distinct results observed in the BELINDA trial compared with the other two. The most relevant differences could be:

- Bridging therapy: In the ZUMA-7 clinical trial, the administration only of steroids (not chemotherapy) was allowed as a bridging treatment between apheresis and product infusion in the CAR-T cell arm, while chemotherapy was allowed as bridging therapy in the other two trials. In total, 36%, 83% and 63% of patients in the ZUMA-7, BELINDA and TRANSFORM trials, respectively, received bridging therapy. These data suggest that the ZUMA-7 trial population may have been more selected and that patients with more aggressive clinical behavior may have been excluded.
- In the BELINDA and TRANSFORM trials, patients randomized to the conventional treatment arm, could cross over to the CAR-T cell arm if the conventional treatment failed, as was the case for half of the patients. The ZUMA-7 trial did not allow crossover, although 56% of patients in the conventional treatment arm received CAR-T cell therapy outside of the clinical trial, so, practically speaking, the crossover rate was similar in the three trials.
- The time between patient inclusion in the clinical trial and CAR-T cell infusion was significantly shorter in the ZUMA-7 and TRANSFORM trials (median 29 and 34 days, respectively) than in the BELINDA trial (median 52 days). This time interval of the BELINDA trial seems excessively long and could have decisively influenced the results. In this trial, 48% of patients required more than one cycle of bridging therapy, including 20 patients who received two different regimens.
- Finally, the definition of EFS (primary objective) was different in the three clinical trials, as mentioned above, providing another potential explanation for the discrepant results.

In summary, the clearly positive results of the ZUMA-7 and TRANSFORM trials have led to CAR-T cell therapy with axi-cel or liso-cel becoming the new standard treatment for patients with refractory or early relapsed DLBCL (within 1 year of completing first-line treatment), after approval by the FDA and EMA.

These clinical trials were designed for patients considered eligible for transplant. Clinical trials have also been carried out with liso-cel (PILOT) [52] and axi-cel (ALYCANTE) [53] as second-line treatment in patients who are not candidates for transplant due to their age, comorbidities or alteration in organ function. The main results of these trials are shown in Table 3. In the PILOT trial, the majority of patients were primary refractory (54%) or in early relapse (21%), but a percentage of patients were in late relapse (25%); in the ALYCANTE trial the presence of primary refractory disease or early relapse was an inclusion criterion. The efficacy and toxicity results of liso-cel and axi-cel in these trials seem comparable to those of the phase 3 trials, which suggests that primary refractory patients or patients with early relapse who are not candidates for transplant, may also benefit from liso-cel or axi-cel. Additionally, liso-cel is approved by the FDA in second-line treatment also for late relapses in patients not candidates for transplant, based on the PILOT trial.

For patients who are not candidates for transplant or CAR-T cell therapy, our recommendation is to include them in a clinical trial whenever possible. Other possible options for these patients would be combinations of polatuzumab vedotin with bendamustine and rituximab (Pola-BR), tafasitamab with lenalidomide or R-GemOx. The results of these regimens will be reviewed below.

## 4. Pola-RB Regimen

Polatuzumab vedotin (PV) is a CD79b-targeted antibody-drug conjugate delivering monomethyl auristatin E, a microtubule inhibitor [56]. PV showed promising efficacy in R/R DLBCL as monotherapy [57] and combined with rituximab [58], yielding ORRs and CR rates of 52–54% and 15–21%, respectively.

A randomized phase 2 clinical trial (*n* = 80) (GO29365) showed that the addition of PV to the R-bendamustine regimen (pola-BR) significantly improves metabolic CR rate (40% vs. 17.5%, *p* = 0.026), PFS (median 9.5 vs. 3.7 months, *p* < 0.001) and OS (median 12.4 vs. 4.7 months, *p* = 0.002). Although the PV arm was associated with higher rates of grade 1–2 neuropathy and grade 3–4 cytopenias, the neuropathy resolved in the majority of patients and cytopenias were not associated with higher rates of infection [47]. Based on this study, the pola-BR regimen was approved by the FDA and EMA for patients with relapsed or refractory DLBCL patients who were not candidates for ASCT. The results of the extension cohort of this clinical trial have recently been published [48]. The cohort comprised 106 patients treated with pola-BR, who exhibited a metabolic CR rate of 39%, and a median PFS and OS of 6.6 and 12.5 months, respectively. The main toxicity was hematological, infectious and neurological, the latter being grades 3–4 in only 2% of patients (Table 3). In the subgroup analysis, better results were obtained in the non-refractory patients (CR, 92%, median PFS and OS, 13 months and not reached, respectively) than in the refractory patients (CR, 40%, median PFS and OS, 6 and 9 months, respectively).

Several real-life studies have been published, which, in general, have reproduced the efficacy results of the GO29365 clinical trial [59,60]. These studies indicate that this regimen may also be effective as a bridging treatment to CAR-T cell therapy, once lymphoapheresis has been performed, since the administration of bendamustine in the months before lymphoapheresis is not recommended due to its prolonged lymphodepletive effect [61,62]. Several clinical trials are investigating the combination of PV with many other agents, including other chemotherapy regimens, such as R-ICE [63] and R-GemOx [64], or bispecific antibodies [65]. They have yielded good preliminary results, although none concerning the efficacy of re-treatment with PV in patients who have received it in the first line.

## 5. Tafasitamab Combined with Lenalidomide

Tafasitamab is an Fc-enhanced, humanized, anti-CD19 monoclonal antibody, that mediates antibody-dependent cellular cytotoxicity and antibody-dependent cellular phagocytosis, and exerts direct cytotoxicity [66]. Single-agent tafasitamab showed promising activity in patients with R/R DLBCL, yielding an ORR and CR rate of 26% and 6%, respectively, with some durable responses (≥12 months in 5/9 responding patients) [67]. On the other side, lenalidomide enhances natural killer cell-meditated, antibody-dependent cellular cytotoxicity with tafasitamab in vitro, providing a rationale for combining the two agents [49].

The combination of tafasitamab with lenalidomide has been approved by the FDA and EMA for patients with R/R DLBCL who are not candidates for ASCT following the good results obtained in the phase 2 L-MIND trial (*n* = 81), which featured high response rates (Table 3), with a median duration of response not reached after a median follow-up of 44 months [49,50,51]. It should be noted that the patient population included in this trial had characteristics of lower risk than what is typical in the context of R/R DLBCL, since primary refractory patients (defined as <PR or progression before 3 months after completing first-line treatment) were excluded, and the vast majority of patients had received only one (50%) or two (43%) previous lines of treatment. Toxicity was manageable, with hematological and infectious toxicity being the most frequent types (Table 3).

A recent real-life study was unable to reproduce the good results of the L-MIND trial [68]. Patients included in this study (*n* = 157) had characteristics of higher risk, such as the presence of primary refractory disease (51% of patients), more comorbidities (33% renal dysfunction), and had been more intensively pre-treated, including CAR-T cell therapy in 28% of patients. In fact, 89% of patients would not have been eligible for the L-MIND trial. Overall response and CR rates were 29% and 17%, respectively, and the median PFS and OS were 2 and 7 months, respectively. The subgroup analysis showed better results in non-refractory patients and in patients who had received fewer lines of treatment, which are possibly the ideal populations to receive this regimen.

## 6. R-GemOx Regimen

R-GemOx (rituximab, gemcitabine, and oxaliplatin) is one of the most used regimens for patients who are not candidates for ASCT. A phase 2 study included 49 patients with relapsed (*n* = 43) or refractory (*n* = 6) DLBCL who were not candidates for ASCT (35% in post-ASCT relapse). After four cycles of R-GemOx, the CR and overall response rates were 44% and 61%, respectively. However, patients with early relapse or progression (<1 year since last treatment vs. >1 year) had significantly lower response rates (36% vs. 81%; *p* < 0.001) and worse PFS (median 3 vs. 10 months, *p* = 0.04). Patients with prior exposure to rituximab also had worse outcomes (response after four cycles 55% vs. 71%, *p* = 0.29; median PFS 4 vs. 11 months, *p* = 0.02). On the other hand, the regimen was well tolerated, with the main grade 3–4 toxicity being hematological (neutropenia and thrombopenia in 73% and 44% of patients, respectively, although only febrile neutropenia in 4% of cycles), infectious (22% of cycles) and neurological (8% of patients) [69]. Therefore, this regimen may allow temporary control of the disease in some patients, with an acceptable toxicity profile for patients who are not candidates for transplant. A more recent real-life study has shown similar results, although the efficacy in refractory patients was very limited, with CR rates and median PFS and OS in non-refractory vs. refractory patients of 50% vs. 10%, 6 vs. 2 months, and 18 vs. 7 months, respectively [70].

## 7. Proposed Second-Line Treatment Algorithm for Patients with DLBCL and Future Perspectives

**Figure 1:**
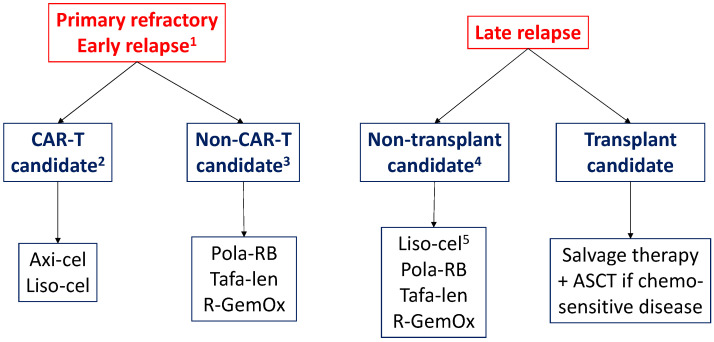
Proposed algorithm for the second-line treatment of DLBCL. ASCT, autologous stem cell transplantation; axi-cel, axicabtagene ciloleucel; DLBCL, diffuse large B-cell lymphoma; liso-cel, lisocabtagene maraleucel; pola-BR, polatuzumab-vedotin with bendamustine and rituximab; R-GemOx, rituximab, gemcitabine, and oxaliplatin; tafa-len, tafasitamab and lenalidomide. ^1^ During the first year following the completion of first-line treatment. ^2^ The selection of treatment (axi-cel or liso-cel) must be individualized based on CAR-T availability and the patient’s characteristics. Liso-cel could be favored in older patients or patients with organ dysfunctions [41]. ^3^ The selection of treatment must be individualized. In countries where CAR-T cell therapy is available in the third, but not the second line, the administration of bendamustine should be avoided in these high-risk patients due to its prolonged lymphodepletive effect [62]. Tafasitamab should also be avoided because it targets CD19, and the potential implications for CAR-T cell therapy if the patient loses CD19, or its expression density decreases, has not been thoroughly studied [71]. For these patients, GemOx +/− R may be a suitable option. The three regimens (pola-RB, tafa-len and R-GemOx) are more efficacious in relapsed than in primary refractory patients. Its inclusion in clinical trials is especially recommended for the latter group of patients. ^4^ The selection of treatment must be individualized according to patient’s characteristics and the toxicity profile of each agent or regimen. ^5^ Liso-cel is approved by the FDA, but not by the EMA, for this indication.

A proposed second-line treatment algorithm for patients with refractory or relapsed DLBCL is shown in Figure 1. CAR-T cell therapy with axi-cel and liso-cel has demonstrated superiority over the standard treatment; therefore, we considered the treatment of choice for patients with high-risk R/R DLBCL. In late relapses, it is necessary to assess whether the patient is a candidate for ASCT. In patients who are not candidates for transplant, the selection of treatment must be individualized based on the patient’s characteristics and their disease.

Anti-CD20-CD3 bispecific antibodies are proving to be highly efficacious in the treatment of R/R DLBCL. They are reviewed in another article in this special issue. Two of them, glofitamab and epcoritamab, have recently been approved by the FDA and EMA for treating patients with R/R DLBCL who were previously treated with two lines. The approvals were based on the results of the phase 2 clinical trials [72,73], in which CR rates of around 40% were described in intensively pre-treated and mostly refractory patients, with no significant differences with respect to prior exposure to CAR-T cell therapy. The remissions seem long-lasting, since most patients who achieve CR maintain it for at least one year. However, the follow-up is still too short to be certain whether this treatment modality is potentially curative, as CAR-T cell therapy has already demonstrated. Therefore, at present, it would be indicated mostly for patients following the failure of CAR-T cell therapy.

On the other hand, polatuzumab vedotin has recently been approved as part of the first-line treatment of patients with DLBCL based on the results of the POLARIX clinical trial [74]. Further, most agents that we currently use in the salvage setting are also being investigated as the first line in phase 3 clinical trials, as a monotherapy, such as axi-cel (ZUMA-23, NCT05371093), or in combination, such as tafasitamab-lenalidomide (frontMIND, NCT04824092), glofitamab (NCT06047080) or epcoritamab (EPCORE DLBCL-2, NCT05578976). The results of these clinical trials could redefine the treatment of R/R patients in the near future. In addition, as more agents and regimens become available, the main challenge will be to sequence the various options and find rational synergistic combinations, guided by patient characteristics, and, preferably, the underlying biological characteristics, based on validated molecular assays and predictive biomarkers.

## Figures and Tables

**Table 1:** Phase 3 clinical trials for patients with relapsed or refractory LBCL.

Patient Characteristics	Design	Efficacy	Comments
**Based on ASCT**			
PARMA [3] 18–60 years Relapsed patients with intermediate or high-grade NHL	DHAP × 2 (*n* = 215): CR/PR (*n* = 109): ABMT vs. DHAP × 2 Phase 3	EFS (5 y), 46% vs. 12% * OS (5 y), 53% vs. 32% *	ABMT is superior to conventional CT in patients with chemosensitive relapse
CORAL [8] 18–65 years R/R DLBCL 61% prior rituximab ECOG 0–1	R-ICE vs. R-DHAP CR or PR: ASCT Phase 3 (PE: ORR) *n* = 396	ORR, 63% vs. 63% ASCT, 50% vs. 54% EFS (3 y), 26% vs. 35% OS (3 y), 47% vs. 51%	No significant differences between R-ICE and R-DHAP
NCIC-CTG LY.12 [9] ≥18 years Aggressive NHL 89% LBCL 67% prior rituximab	(R)-GDP vs. (R)-DHAP CR or PR: ASCT Phase 3 (PE: ORR and ASCT rate) *n* = 619	ORR, 45% vs. 44% ASCT, 52% vs. 49% EFS (4 y), 26% vs. 26% OS (4 y), 39% vs. 39%	No differences in efficacy, but R-GDP is less toxic than R-DHAP
ORCHARRD [10] ≥18 years R/R DLBCL 100% prior rituximab	R-DHAP vs. O-DHAP CR or PR: ASCT Phase 3 (PE: PFS) *n* = 447	ORR, 42% vs. 38% ASCT, 37% vs. 33% PFS (2 y), 26% vs. 24% OS (4 y), 38% vs. 41%	No benefit of changing rituximab to ofatumumab
**CAR-T cell therapy vs. ASCT**			
ZUMA-7 [11,12] ≥18 years Early relapsed ^#^ or refractory LBCL Eligible for ASCT *n* = 359	Axi-cel vs. SOC ^&^ No protocol-specified crossover Steroid-only bridging in axi-cel arm (36%) Turnaround ^€^ 29 days Phase 3 (PE: EFS)	EFS (2 y), 40% vs. 16% * Median, 8.3 vs. 2.0 mo ORR, 83% vs. 50% * CR, 65% vs. 32% OS (4 y): 55% vs. 46% * Median, NR vs. 31.1 mo	Axi-cel is superior to SOC as second-line for patients with early R/R LBCL
BELINDA [13] ≥18 years Early relapsed ^#^ or refractory LBCL Eligible for ASCT *n* = 332	Tisa-cel vs. SOC ^&^ Crossover allowed (50%) Bridging chemo in tisa-cel arm (83%) Turnaround ^€^ 52 days Phase 3 (PE: EFS)	EFS, Median, 3.0 vs. 3.0 mo ORR, 46% vs. 42% CR, 28% vs. 27%	Tisa-cel is not superior to SOC as second-line therapy for patients with early R/R LBCL
TRANSFORM [14,15] ≥18 years Early relapsed ^#^ or refractory LBCL Eligible for ASCT *n* = 184	Liso-cel vs. SOC ^&^ Crossover allowed (63%) Bridging chemo in liso-cel arm (63%) Turnaround ^€^ 34 days Phase 3 (PE: EFS)	EFS (18 mo), 53% vs. 21% * Median, NR vs. 2.4 mo ORR, 87% vs. 49% * CR, 74% vs. 43% * OS (18 mo): 73% vs. 61% Median, NR vs. 29.9 mo	Liso-cel is superior to SOC as second-line treatment for patients with early R/R LBCL

* Statistically significant difference. ^#^ Equal or less than 12 months of first-line treatment. ^&^ Investigator-selected platinum-based immunochemotherapy × 2–3 cycles, followed by HDT-ASCT in responders. In the BELINDA trial, a second regimen was allowed. ^€^ Median time from registration to CAR-T infusion. Abbreviations: ASCT, autologous stem cell transplantation; axi-cel, axicabtagene ciloleucel; CAR-T, chimeric antigen receptor T-cells; CR, complete response; CT, chemotherapy; DHAP, dexamethasone, citarabine, and cisplatin; DLCBG, diffuse large B-cell lymphoma; EFS, event-free survival; GDP, gemcitabine, dexamethasone, and cisplatin; ICE, ifosfamide, carboplatin, and etoposide; LCBG, large B-cell lymphoma; liso-cel, lisocabtagene maraleucel; mo, months; NHL, non-Hodgkin’s lymphoma; NR, not reached; O, ofatumumab; ORR, overall response rate; OS, overall survival; PE, primary endpoint; PR, partial response; R, rituximab; R/R, refractory or relapsed; SOC, standard of care; tisa-cel, tisagenlecleucel; y, years.

**Table 2:** Efficacy of salvage regimens in patients with DLBCL pre-treated with rituximab (phase 3 trials).

Regimen	*n*	CR (%)	ORR (%)	ASCT (%)	PFS/EFS	OS
R-DHAP [10] *	223	22 ^#^	42 ^#^	37	26% (2 y)	38% (2 y)
O-DHAP [10] *	224	15 ^#^	38 ^#^	33	24% (2 y)	41% (2 y)
R-ICE / R-DHAP ** [8]	244	ND	51 ^##^	NA	21% (3 y)	40% (3 y)
R-GDP/ R-DHAP *** [9]	318	16 ^##^	46 ^##^	52	22% (4 y)	35% (4 y)

* ORCHARRD trial. ** Subgroup of patients pre-treated with rituximab in the CORAL trial (pooled results for R-ICE and R-DHAP regimens). *** Subgroup of patients pre-treated with rituximab in the NCIC Clinical Trials Group Study LY.12 trial (pooled results for R-GDP and R-DHAP regimens). ^#^ Cheson 2007 criteria [17]. ^##^ Cheson 1999 criteria [18]. Abbreviations: ASCT, autologous stem cell transplantation; CR, complete response; DHAP, dexamethasone, citarabine, and cisplatin; DLBCL, diffuse large B-cell lymphoma; EFS, event-free survival; GDP, gemcitabine, dexamethasone, and cisplatin; ICE, ifosfamide, carboplatin, and etoposide; NA, not available; O, ofatumumab; OS, overall survival; ORR, overall response rate; PR, partial response; R, rituximab; y, years.
